# What resources do elderly people choose for managing their symptoms? Clarification of rural older people’s choices of help-seeking behaviors in Japan

**DOI:** 10.1186/s12913-021-06684-x

**Published:** 2021-07-03

**Authors:** Ryuichi Ohta, Mikiya Sato, Yoshinori Ryu, Jun Kitayuguchi, Tetsuhiro Maeno, Chiaki Sano

**Affiliations:** 1 Community Care, Unnan City Hospital, 96-1 Iida Daito-cho, Unnan City, Shimane Prefecture Japan; 2 Department of Primary Care and Medical Education, Graduate School of Comprehensive Human Sciences, University of Tsukuba, Tsukuba, Japan; 3 Health Services Development and Research Center, University of Tsukuba, Tsukuba, Japan; 4 Health Services Center, Occupational Safety and Health Department, Human Resources Group, Sumitomo Heavy Industries, Ltd., Tokyo, Japan; 5 Physical Education and Medicine Research Center Unnan, Unnan, Shimane Prefecture Japan; 6 Faculty of Medicine, University of Tsukuba, Tsukuba, Japan; 7 Department of Community Medicine Management, Faculty of Medicine, Shimane University, 89-1 Enya cho, Izumo, Shimane Prefecture Japan

**Keywords:** Elderly patients, Help-seeking behavior, Japan, Lay care, Professional care

## Abstract

**Background:**

Appropriate help-seeking behavior (HSB) that involves lay and professional care may moderate the usage of medical resources and promote good health, especially among the rural elderly. However, there is little evidence regarding the rural elderly’s HSB choices for mild symptoms. Therefore, this study attempts to bridge this gap.

**Methods:**

The participants were patients living in rural areas and over the age of 65, who attended Japanese clinics and general hospitals. In Phase 1, monthly diaries and one-on-one interviews about their mild symptoms and HSB were used to establish HSB items and assess its content validity. Content analysis helped determine the items. In Phase 2, participants were asked to complete the list to measure HSB. The answers to the list and HSB mentioned in the diaries were compared to evaluate concurrent validity. Retests were conducted to examine the content’s reliability and test-retest reliability.

**Results:**

Phase 1 included 267 participants (average age = 75.1 years, standard deviation [SD] = 4.3; 50.1% male). The diary collection rate was 97.6%. Of the participants, 70.4% used lay care and 25.4% used professional care. Content analysis identified eight types of lay care and four types of professional care. Phase 2 included 315 participants (average age = 77.7 years, SD = 8.27; 46.0% male). In terms of validity, the results of the list and the diaries were correlated (Spearman r 0.704; *p* < 0.001). The most common behavior with mild symptoms was consulting with primary care physicians, followed by self-care and using home medicine. The test-retest reliability for mild symptoms found kappa values of 0.836 for lay care and 0.808 for professional care.

**Conclusions:**

The choices of HSB for mild symptoms clarified identified in this study have high validity and reliability. Therefore, it can be used to assess the relationships between HSB and health conditions and the effectiveness of health promotion on rural older people’s HSB.

**Supplementary Information:**

The online version contains supplementary material available at 10.1186/s12913-021-06684-x.

## Background

Help-seeking behavior (HSB) is a human behavior that sustains health and involves seeking treatment for symptoms. It is an essential behavior for maintaining peoples’ health; ideally, each person should have appropriate HSB. This requires people to assess their symptoms and its severity, use lay care to manage these symptoms, followed by professional care as needed [1]. Suitable HSB can connect people with appropriate care and enhance peoples’ health in ways such as better quality of life [2]. A balance of lay care and professional care is important for appropriate HSB [3]. Lay care is provided by lay people, or those who have received no formal training and are not paid, such as self-care and care from relatives, friends, and self-help groups, while professional care refers to care provided by trained paid professionals, usually in a formal setting [4]. The efficient usage of lay and professional care can reduce people’s inappropriate HSB [5, 6].

HSB varies relative to healthcare resources. In urban areas, there are many medical resources, allowing for choice between various medical and healthcare professionals [1]. However, citizens in rural areas lack adequate resources for medical care, leading to inappropriate usage and inappropriate treatments such as medical care for mild symptoms [7]. Limited education among people living in rural areas may also affect HSB [8–10]. In addition, rural HSB needs to be improved by providing information and educational interventions of HSB from multiple perspectives as these people may also lack information about medical issues [7, 11]. Although the Internet and social media have advanced, they are primarily used by young and middle-aged people. The elderly tend to obtain information from television and newspapers [12, 13]. A lack of information may lead to inappropriate HSB especially for mild symptoms [14]. Furthermore, it results in excessive usage of medical resources and undesirable results for citizens’ health [2].

Rural older people’s HSB for mild symptoms can be improved by continuously providing them with accurate information about their symptoms and healthcare [15]. In aging societies, issues in older people’s HSB can critically affect health care. Management of older people’s mild symptoms should be facilitated effectively. Therefore, it is crucial to assess their HSB for mild symptoms to suggest modifications. There is a need for a comprehensive investigation of rural older people’s HSB choices that are reliable and valid to evaluate their HSB for mild symptoms [16]. Furthermore, HSB for mild symptoms can be assessed in terms of its relationship with patients’ demographic characteristics. Currently, no study has comprehensively clarified rural older people’s HSB that are reliable and valid. Japan, one of the aged countries in the world, has critical issues regarding HSB for mild symptoms [15, 17]. Understanding the choices of rural older people’s HSB in Japan can be beneficial for other aging countries in Asia with cultural similarity. Furthermore, through comparisons of HSB of older adults from different countries, relationships between HSBs and cultural and social conditions can be investigated. Therefore, this study aimed to create comprehensive choices that can accurately assess rural older people’s HSB for mild symptoms.

## Methods

### Setting

Unnan City, located in southeast Shimane Prefecture, Japan, is primarily covered by forest and is one of the most rural cities in Japan. A 2017 survey revealed the total population was 38,882 (consisting of 18,720 males and 20,162 females) and that the percentage of people over the age of 65 was 37.82%; this is estimated to reach 50% by 2025 [15]. Kakeya and Tai Clinic are rural clinics in Kakeya and Yoshida Town, the towns situated in the most northern part of Unnan City. Both clinics are about 30 km from Unnan City Hospital, the only general hospital in the city. Kakeya Clinic has five registered physicians, two nurses, and no admission facilities. Tai Clinic has three registered physicians, two nurses, and no admission facilities. All the physicians in both clinics are family medicine specialists. At the time of this study, Unnan City Hospital had 281 beds comprising 160 acute care beds, 43 comprehensive care beds, 30 rehabilitation beds, and 48 chronic care beds. There were 14 medical specialties, and the nurse-to-patient ratio was 1:10 in acute care, 1:13 in comprehensive care, 1:15 in rehabilitation, and 1:25 in chronic care [10].

### Participants

All participants in this study were aged over 65 years and visited Kakeya and Tai Clinic and Unnan City Hospital for regular checkups for chronic diseases once or twice a month. The total number of participants were 169 for the clinics and 146 for the hospital’s general medicine division. Their regular checkup for chronic diseases was supported by the Japanese National Health Insurance, whereby older adults aged less than 70, 70–74, and over 75 years had to pay 30, 20, and 10% of the total cost, respectively [18]. All participants lived in Unnan City. They were informed of this study via wall posters and physicians in the clinics and hospital; an explanation of the purpose of the research was included in the list and monthly health diary. The inclusion criteria were: being over the age of 65; regularly visiting the clinics or the hospital; and being able to read, write, and hear properly. The exclusion criteria were patients who could not read, write, and hear properly to answer the instrument, including patients with dementia and cognitive impairment.

### Phase 1: instrument development

#### Monthly health-check diary

The diaries consisted of a space for noting the date, patient’s blood pressure, existence of acute symptoms, types of symptoms, and how they managed the symptoms (i.e., HSB) (Supplementary file 1). For 1 month, respondents who agreed to participate in phase 1 checked whether they had symptoms at the end of each day. The clinic nurses collected the diary pages when the participants visited the clinic 1 month later. If there were unclear terms in their diary, the clinic nurses checked their symptoms and their approach to the symptoms in depth and wrote this on the diary page in red ink. This phase of the study was conducted from September to November 2019.

#### One-on-one interviews and a review of previous studies to confirm the content validity

To create a valid HSB list, participants’ HSB was first transcribed based on the diaries. Next, based on previous studies [16, 19], purposive sampling was used to select 39 participants (11 from Kakeya Clinic, 9 from Tai Clinic, and 19 from the hospital) from among those who had completed their respective diaries. Participants who were motivated to talk about their symptoms and high education were sampled, to describe their symptoms clearly. The first author, a family physician, interviewed these participants about their potential HSB for mild symptoms, to confirm the concurrent validity, until data saturation was reached. The interviews were performed in the clinics and the hospital, taking into consideration each participant’s privacy. The interview guide consisted of four questions based on a previous HSB study [19]: “What kind of symptoms do you have in your usual lives?” “How do you act when you have mild bodily symptoms?” “Why do you act so?” and “Please describe your concrete experiences.” (Supplementary file 2) To define mild symptoms, a previous study on HSB was referred to [16]. Based on these findings, participants were presented examples of mild symptoms: mild fatigue, flu-like symptoms, joint pain, back pain, and mild headache [16]. The contents of the interviews were then recorded and transcribed verbatim, so that the first and second authors could review the interview and diary transcriptions through the content analysis method [20]. Through this process, the list of options of potential HSB was finally constructed (Supplementary file 3). This phase of the study was conducted from December 2019 to January 2020.

### Phase 2: testing the validity and reliability of the HSB list

The participants who agreed to participate in phase 2 were given the constructed list of options for their potential HSB. Multiple answers were allowed for each question. By comparing the answers on the list and the results for the HSB in the monthly diaries, the validity of the list was assessed. Lists were completed in the waiting room at the clinic and then collected by nurses. To confirm the reliability of the HSB list, all participants were provided with the constructed HSB list twice, ensuring test-retest reliability. The interval between the two rounds of list completion was 1 month. The participants’ data were also extracted from the clinic’s electronic medical record and a questionnaire including age, sex, work conditions, exercise habits, eating habits, sleeping habits, smoking, habitual alcohol drinking, educational level, living conditions, social support [16], social capital (using a 10-point Likert scale: can rely on neighbors in communities to completely not rely on neighbors in communities) [21], socioeconomic state and health literacy (HLS-14) [22], and diseases. This phase of the study was conducted in February 2020.

### Data analysis

The first and second authors performed the content analysis [20]. Initially, the authors individually read the contents of the interview transcripts in depth while referring to the contents of the health-check diaries. After reading them, they generated the content regarding the HSB from the scripts. Symptoms from the health-check diaries were categorized based on the International Classification of Primary Care-2. Their real HSB based on their diaries and the HSB based on the constructed questionnaire were categorized into two groups based on a previous study: lay care and professional care. Regarding lay care, whether or not the patients’ real and potential HSB were correlated was analyzed using the chi-squared test and Spearman r, and statistical significance was set with *p* < 0.05. Regarding professional care, the participants’ real HSBs could be affected by their symptoms’ duration and severity, and the participants with only self-limited symptoms may not use professional care [19]. Hence, correlation between patients’ real and potential HSBs was revealed by the percentage agreement between the results of the lists and diaries. The results of the repetition of the HSB list for lay and professional care were analyzed using Cohen’s kappa statistic. The other independent variables were categorized binomially: sex (male = 1, female = 0), work (in employment = 1, not employed = 0), smoking (yes = 1, no = 0), alcohol drinking (yes = 1, no = 0), higher educational level (more than graduation from high school = 1, no = 0), living conditions (with family = 1, living alone = 0), higher social support (having or relative having = 1, not relatively having or not having = 0), higher social capital (high [10 to 16] = 1, low [5 to 1] = 0), and higher socioeconomical state (high [rich, relatively rich, or not poor] = 1, low [relatively poor or poor] = 0). Regarding HLS-14, the participants were divided into two groups according to a total HLS of above or below the median. Based on kinds of diseases, a Charlson Comorbidity Index (CCI) score was calculated for each participant to assess their severity of medical conditions. This index measures the severity of patients’ medical conditions as it relates to the possibility of admissions and mortality [23]. Cases with missing data were excluded from the analysis. Regarding the sample size calculation, it was estimated that at 80% statistical power and 5% type 1 error, 238 participants would be needed to detect a significant validity and reliability.

### Ethical considerations

Participants were informed that the data collected in this study would be used only for research purposes. They were also informed about the aims of this research, how the data would be disclosed, and that their personal information would be protected. Thereafter, they provided written informed consent.

### Ethics approval

The study was conducted in accordance with the principles of the Declaration of Helsinki and approved by Unnan City Hospital’s Clinical Ethics Committee (approval number: 20190033).

## Results

### Phase 1: instrument development

A total of 267 respondents (121 from the clinics and 146 from the hospital) agreed to participate in Phase 1. The participants’ average age was 79.3 years (standard deviation [SD] = 6.8), and 38.2% were male. The collection rate of the diaries was 98.1% (262/267). A total of 214 participants had experienced symptoms. The total number of symptoms was 262. The most common was joint pain (L20), followed by headache (N01) and fatigue (A04) (Table 1). Of the participants, 86.3% (226/262) used lay care and 13.7% (36/262) used professional care to address these symptoms. Eighty-four codes were derived for the approaches from the diary sheets, and the most common code found was self-care (132/262), followed by self-medication (72/262), using primary care (32/262), and using complementary medicine (21/262). In the content analysis, eight subcategories of lay care and four for professional care were created. The lay care category consisted of doing nothing, self-care (changing lifestyles, sleeping, resting, and taking a bath), seeking information, consulting family and friends, consulting community members, using complementary medicine, using home medicine, and buying over-the-counter drugs. The category of professional care included consulting pharmacists, consulting primary care physicians, visiting medical institutions (other than primary care physicians), and visiting general hospital emergency rooms (including calling for an ambulance) (Table 2).

**Table 1:** Codes of the symptoms and classification using the International Classification of Primary Care-2

Ranking	Code number	Percentage	Category	ICPC-2
1	53	20.2	Joint pain	L20
2	45	17.2	Headache	N1
3	36	13.7	Fatigue	A4
4	26	9.9	Itchiness	S2
5	20	7.6	Muscle pain	L18
6	16	6.1	Diarrhea	D11
7	15	5.7	Back pain	L3
8	14	5.3	Vertigo	N17
9	7	2.7	Numbness	N5
10	6	2.3	Abdominal pain	D6
10	6	2.3	Insomnia	P6
12	5	1.9	Common cold	R74
	13	5.0	Others	

Others = chest pain, palpitation, muscle spasm, difficulty in hearing and seeing, chill.

*ICPC-2* International Classification of Primary Care-2

**Table 2:** Items of the help-seeking behavior list and definition of each item

Category	HSB	Definition
Lay care	Doing nothing	No action
	Self-care (sleeping, resting, taking a bath)	Moderating symptoms by changing usual behaviors
	Seeking information	Collecting information about symptoms
	Consulting family and friends	Asking for help from family and friends
	Consulting community members	Asking for help from community members
	Using complementary medicine	Using chiropractic treatment or osteopathy
	Using home medicine	Using medicine that is present in the home
	Using OTC drugs	Buying OTC drugs in drug stores
Professional care	Consulting pharmacists	Asking pharmacists for treatment
	Consulting primary care physicians	Visiting primary care physicians for treatment
	Visiting medical institutions other than primary care physicians	Visiting medical institutions excluding primary care physicians for treatment
	Visiting the emergency room of general hospitals (including calling for an ambulance)	Going to emergency rooms of general hospitals for treatment

*HSB* Help-seeking behavior, *OTC* Over-the-counter

### Phase 2: testing the validity and reliability of the HSB list

The number of participants who responded to the HSB list was 315 (169 from the clinics and 146 from the hospital) with a 100% collection rate. The participants’ average age was 77.7 years (SD = 8.2), and 46.0% were male. Regarding HLS-14, the median score was 49. The CCI scores for 33.9% (107/315) of the participants were greater than 5 (Table 3). In terms of validity, the results of the list and the diaries were correlated (Spearman r 0.704; *p* < 0.001). The most common behavior with mild symptoms, during this phase, was consulting with primary care physicians, followed by self-care and using home medicine (Table 4). The test-retest reliability for mild symptoms found kappa values of 0.836 for lay care and 0.808 for professional care.

**Table 3:** Demographic characteristics of the participants

Variable	***N*** = 315
Age (years), mean (*SD*)	77.71 (8.27)
Sex (males), %	46.0
Smoking, %	40 (12.7)
Work, %	140 (54.4)
Living alone, %	41 (13.0)
Higher education, %	149 (47.3)
Higher social capital, %	168 (53.3)
Higher social support, %	229 (72.7)
Higher SES, %	259 (82.2)
Higher HL, %	159 (50.4)
Charlson Comorbidity Index, number (%)	
Score = 2	48 (15.2)
Score = 3	62 (19.7)
Score = 4	98 (31.1)
Score = 5	67 (21.3)
Score = 6	24 (7.6)
Score = 7	12 (3.8)
Score = 8	4 (1.3)
Score≧5	107 (33.9)
Diseases, number (%)	
Hypertension	293 (95.2)
Dyslipidemia	254 (80.6)
Diabetes mellitus	53 (16.8)
Chronic kidney disease	60 (19.0)
Heart failure	31 (9.8)
Cerebral vascular disease	21 (6.7)
Cancer	19 (6.0)
Hepatic disease	15 (4.8)
COPD	13 (4.1)
Asthma	12 (3.8)
Myocardial infarction	8 (2.5)

*SD* Standard deviation, *COPD* Chronic obstructive pulmonary disease, *SES* Socioeconomic state, *HL* Health literacy

**Table 4:** Prevalence of help-seeking behavior for mild symptoms

HSB	Mild symptoms	
	***N***	Percentage
Doing nothing	10	3.1
Self-care (changing lifestyles, sleeping, resting, taking a bath)	146	46.3
Seeking information	42	13.3
Consulting family and friends	126	40.0
Consulting community members	10	3.2
Using complementary medicine	27	8.6
Using home medicine	129	41.0
Using OTC drugs	97	30.8
Consulting pharmacists	15	4.8
Consulting primary care physicians	227	72.1
Visiting medical institutions other than primary care physicians	19	6.0
Visiting the emergency room of general hospitals (including calling for an ambulance)	17	5.4

*HSB* Help-seeking behavior, *OTC* Over-the-counter

## Discussion

This is the first study to clarify rural older people’s choices regarding lay and professional care in their HSB and to verify its validity and reliability. The two phases of the study were designed to confirm content validity through various forms of data collection and testing. This list can provide a stepping-stone for further research regarding HSB of individuals and health outcomes.

Content validity of the list was examined through diaries of symptoms, review of previous studies, and one-to-one interviews. Content validity is vital for lists because it ensures the list accurately evaluates what the study intends to measure, which this study accomplished through a survey assessing patients’ real HSB, providing real situational evidence to various HSB choices [16, 19]. In this study, 33.9% of the participants had a disease severity score greater than 5 on the CCI, and most patients were not deemed critical. Thus, the severity of diseases in this study was similar to those reported in previous studies [16, 19]. Existing studies were also used when developing list items because they discuss the categorization and concepts of the severity of symptoms [16, 19]. Furthermore, the one-to-one semi-structured interviews deepened the understanding of the behaviors, allowed for further inquiry into possible behaviors, and confirmed behaviors suggested in the analysis of the diaries and research reviews.

The analysis exemplified the list’s high concurrent validity and reliability by comparing the real activities and results of the list and test-retest. Through this comparison, a strong correlation was found between the two; therefore, the list has high validity [19]. As the participants were allowed to simultaneously choose multiple items in the list, the correlation and test-retest were performed for both lay and professional care, and high reliability and validity in both categories were found [24].

The patients’ symptoms in this study were similar to those in previous studies, but the rate of using primary care physicians in this study was higher, and the rate of self-care was lower than those reported in studies conducted in other countries [16, 25, 26]. This finding may be attributable to the medical conditions of older patients in developed countries, especially in rural Japan [15]. Older patients may have difficulties managing their symptoms because of their lower health literacy [14]. In other countries, older people live with their relatives and can consult them about how to approach their symptoms, so older people’s HSB may depend on their families, relatives, and neighbors [1, 11]. Meanwhile, in Japan, as rural older people are isolated and live only with their partners without enough help, this can lead to a trend of depending on medicine. The rate of self-care and self-medication with mild symptoms (using home medicine and over-the-counter medicine) was low.

The low rate of consulting community members and pharmacists may reflect the trend among older people’s changing behaviors, which are affected by ageism. Older people may experience vague or irregular symptoms in combination with aging, which means that there are unavoidable symptoms accompanied by prejudices and ageism [25, 27–29]. Because of ageism, older people tend to deem their symptoms unmanageable and HSB not meaningful, which may further isolate them from the society [29]. In aged societies, health promotion strategies should focus on not only young-to-middle-aged adults but also older people, to make healthcare systems sustainable. To do so, older people should be included in societies, which would improve their health outcomes, such as disability-free life expectancy [30]. Future studies should investigate the relationship between ageism and older people’s HSB, and the effect of including older people in communities on their health conditions.

Furthermore, HSB can affect older people’s health indicators, such as quality of life and self-rated health. These health outcomes can be affected by their health and social conditions such as chronic diseases of various severities, healthcare accessibility, and treatment affordability. Previous studies have shown a relationship between HSB and clinical outcomes, such as brain stroke and heart failure, focusing on acute and emergency situations [12, 27, 28]. However, there has been no study on the relationship between HSB for mild symptoms and health outcomes, as well as aging [31, 32]. Future research should investigate this relationship and interventions for improving older people’s HSB should be developed.

There are several limitations to this study. Regarding validity, although the content validity was clarified, the concurrent validity for professional care with mild symptoms was not clarified. The various presentations of mild symptoms, such as durations and timing, affect HSBs, making identifying real HSBs difficult. HSB can depend on the individual’s situation. Living environment can change HSB in terms of three factors: accessibility, availability, and affordability [33, 34]. The setting of this study was rural, so these three elements may have been low, potentially causing minimal use of primary care and other medical institutions. As the list is comprehensive and contains various behaviors, it can be used in different settings, clarifying multiple types of HSB. Another limitation is the difference in health insurance between countries. Japan’s medical system is a free access system; thus, Japanese people can access medical institutions anytime and anywhere [35]. When applying this list to other countries’ settings, other possible behaviors should be considered based on the local medical systems. Furthermore, the sampling was limited to rural settings and likely biased because of participants’ varying motivation to participate in the one-on-one interviews. Future studies should investigate other contexts both quantitatively and qualitatively.

## Conclusions

This study clarified rural older people’s HSB choices for mild symptoms. The choices of HSB for mild symptoms identified in this study have high validity and reliability in the Japanese context, and it can be used in various contexts to assess people’s HSB for mild symptoms.

## Supplementary Information

**Additional file 1.**

**Additional file 2.**

**Additional file 3.**

## Data Availability

The datasets used and/or analyzed during this study are available upon reasonable request from the corresponding author.

## Abbreviation

HSB: Help-seeking behavior
