# Supplementary material for: What resources do elderly people choose for managing their symptoms? Clarification of rural older people’s choices of help-seeking behaviors in Japan
Source: BMC Health Serv Res. 2021 Jul 3;21:640. doi: 10.1186/s12913-021-06684-x (PMC8254357; doi:10.1186/s12913-021-06684-x)
Supplement: Supplementary file 1 — Additional file 1. [file 12913_2021_6684_MOESM1_ESM.pdf]

Name（　　　　　　　　　　　　　　　　　）

Diary of your symptoms　～Please check your symptoms everyday～

date	/	/	/	/	/	/	/	/	/	/	/	/	/	/
Blood pressure	/	/	/	/	/	/	/	/	/	/	/	/	/	/
Do you have any symptoms?	Yes No	Yes No	Yes No	Yes No	Yes No	Yes No	Yes No	Yes No	Yes No	Yes No	Yes No	Yes No	Yes No	Yes No
Concrete symptoms														
How to approach?														

日付	/	/	/	/	/	/	/	/	/	/	/	/	/	/
血圧	/	/	/	/	/	/	/	/	/	/	/	/	/	/
Do you have any symptoms?	Yes No	Yes No	Yes No	Yes No	Yes No	Yes No	Yes No	Yes No	Yes No	Yes No	Yes No	Yes No	Yes No	Yes No
Concrete symptoms														
How to approach?														
