# Supplementary material for: What resources do elderly people choose for managing their symptoms? Clarification of rural older people’s choices of help-seeking behaviors in Japan
Source: BMC Health Serv Res. 2021 Jul 3;21:640. doi: 10.1186/s12913-021-06684-x (PMC8254357; doi:10.1186/s12913-021-06684-x)
Supplement: Supplementary file 2 — Additional file 2. [file 12913_2021_6684_MOESM2_ESM.pdf]

Interview guide

Inquiring the contents of help-seeking behaviors of citizens

<u>What is help-seeking behavior?</u>

Human behaviors that sustain health and involves seeking treatment for symptoms when people have symptoms and concerns.

We would like to interview you to know your usual behaviors to your symptoms to know rural older people's help-seeking behaviors to mild symptoms.

The explanation

-The interview's duration is about 20 minutes.

-The contents of the interview were used in this research.

-The interview contents are used for research only.

-The content of the interview will be recorded and transcribed verbatim. The recorded data will be discarded after use to protect personal information.

-If you have any inconvenience, you can withdraw at any time, and you will not suffer any medical disadvantage.

-In the unlikely event that you suffer a disadvantage, you will immediately stop participating in the research and will not use the data at all.

-Participants' information is based on national guidelines, including the fact that privacy and human rights are adequately protected. If you have any questions or concerns regarding this request, please contact the following: Unnan City Hospital Community Care Department: +81854-47-7500

Interview guide

I will ask the following four questions:

1. "What kind of symptoms do you have in your usual lives?"
2. "How do you act when you have mild bodily symptoms?"
3. "Why do you act so?"
4. "Please describe your concrete experiences."

Ryuichi Ohta

Unnan city hospital
