# Supplementary material for: What resources do elderly people choose for managing their symptoms? Clarification of rural older people’s choices of help-seeking behaviors in Japan
Source: BMC Health Serv Res. 2021 Jul 3;21:640. doi: 10.1186/s12913-021-06684-x (PMC8254357; doi:10.1186/s12913-021-06684-x)
Supplement: Supplementary file 3 — Additional file 3. [file 12913_2021_6684_MOESM3_ESM.pdf]

Check list of Help-seeking behavior

I would like to ask you about how to deal with the symptoms you feel in your daily life.

If you have mild symptoms (fatigue, common cold, joint pain, back pain, headache, anxiety. ext), how do you act? Please choose your behavior as following (you can choose multiple behaviors).

- □1 Doing nothing
- □2 Self-care (sleeping, resting, taking a bath)
- □3 Seeking information
- □4 Consulting family and friends
- □5 Consulting community members
- □6 Using complementary medicine
- □7 Using home medicine
- □8 Using over-the-counter drugs
- □9 Consulting pharmacists
- □10 Consulting primary care physicians
- □11 Visiting medical institutions other than primary care physicians
- □12 Visiting the emergency room of general hospitals (including calling for an ambulance
